# Supplementary material for: Molecular-Scale Investigations Reveal the Effect of Natural Polyphenols on BAX/Bcl-2 Interactions
Source: Int J Mol Sci. 2024 Feb 20;25(5):2474. doi: 10.3390/ijms25052474 (PMC10931803; doi:10.3390/ijms25052474)
Supplement: Supplementary file 1 [file ijms-25-02474-s001.zip › ijms-2844332-supplementary.pdf]

Molecular-Scale Investigations Reveal the Effect of Natural Polyphenols on BAX/Bcl-2 Interactions

Heng Sun, Fenghui Liao, Yichen Tian, Yongrong Lei, Yuna Fu and Jianhua Wang *

Supporting information

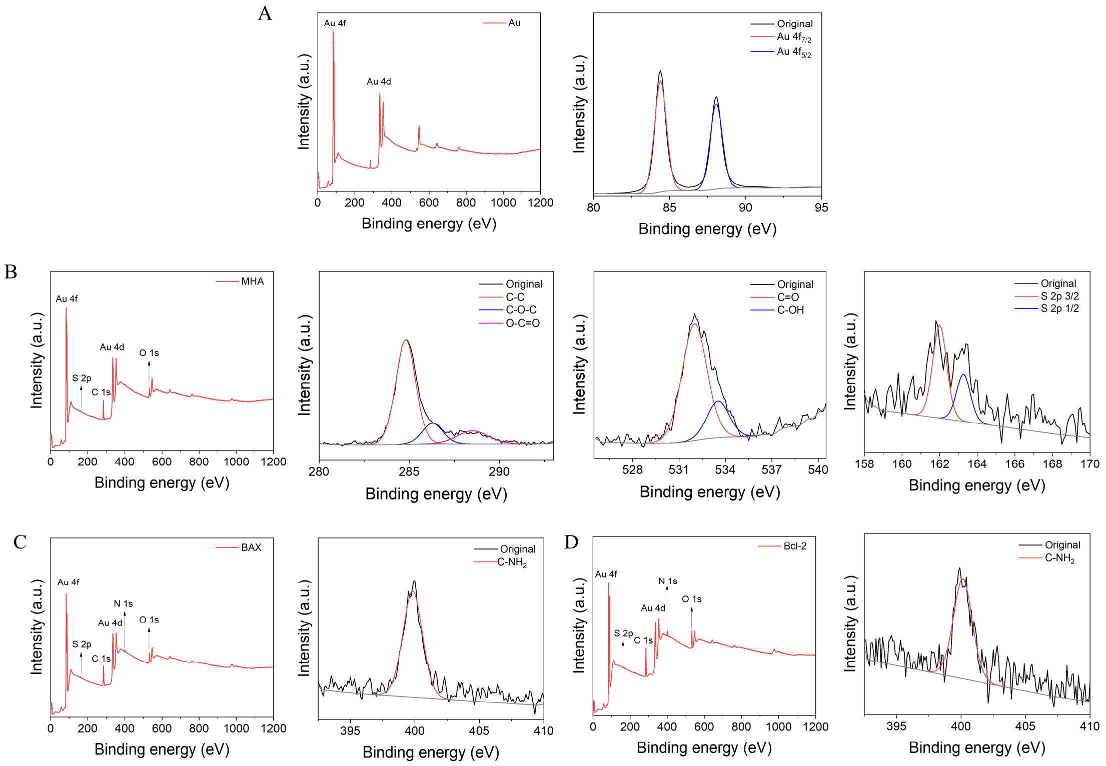

Figure S1. The XPS characterization of immobilized proteins. (A) XPS survey spectrum of Au substrate and Au 4f spectrum. (B) XPS survey spectrum of MHA modified gold substrate, and high-resolution spectrum of C 1s, O1s and S 2p, respectively. (C) XPS survey spectrum of BAX modified MHA substrate, and high-resolution spectrum of N 1s. (D) XPS survey spectrum of Bcl-2 modified MHA substrate, and high-resolution spectrum of N 1s.

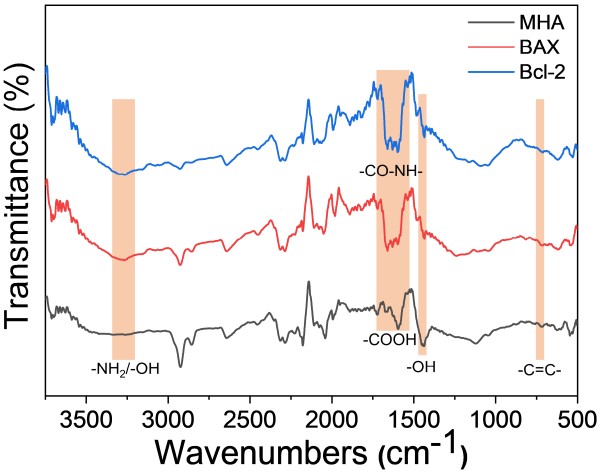

Figure S2. FT-IR spectrum of substrate before and after modification with BAX or Bcl-2 proteins.

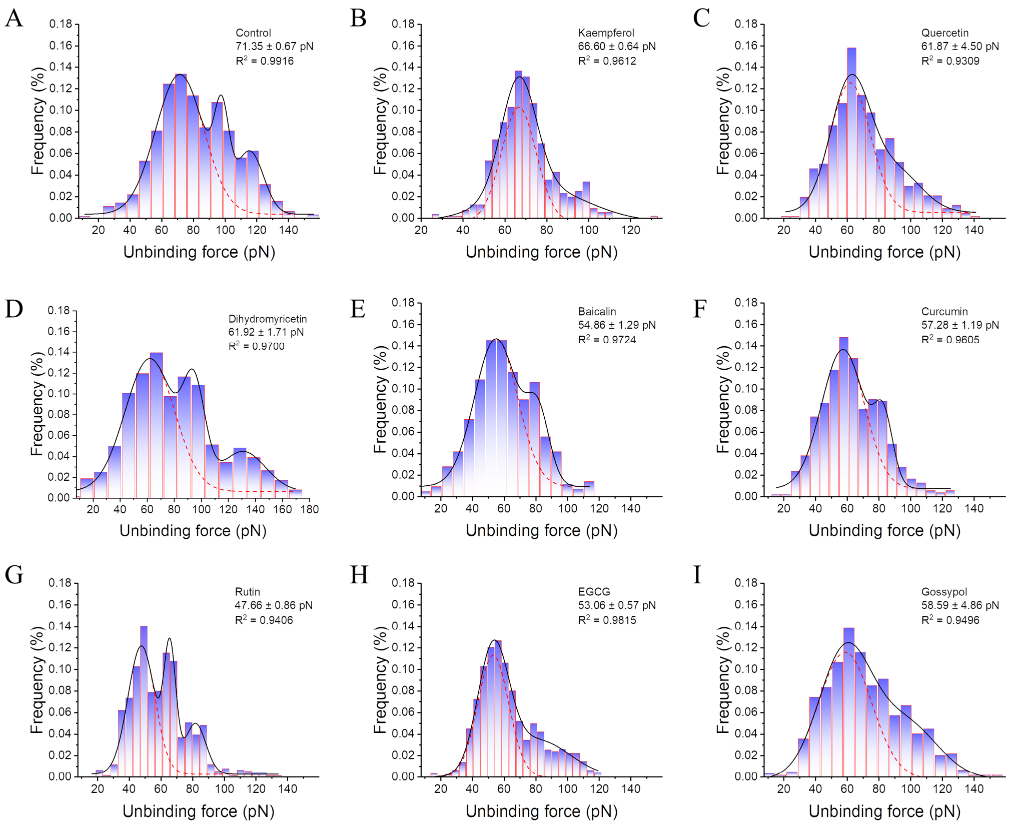

Figure S3. Frequency distribution of unbinding forces between BAX/Bcl-2 pairs under the control and polyphenols and the corresponding multipeak Gaussian fitting curves were measured at a loading rate of 10 nN/s.

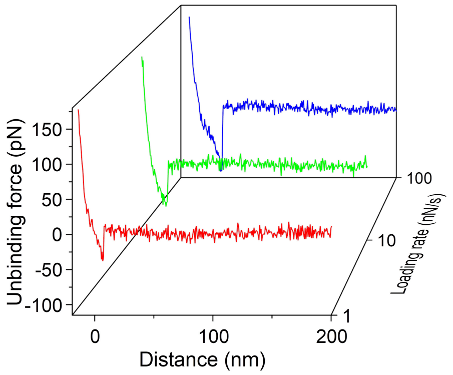

Figure S4. Representative force-distance curves recorded at different loading rates.

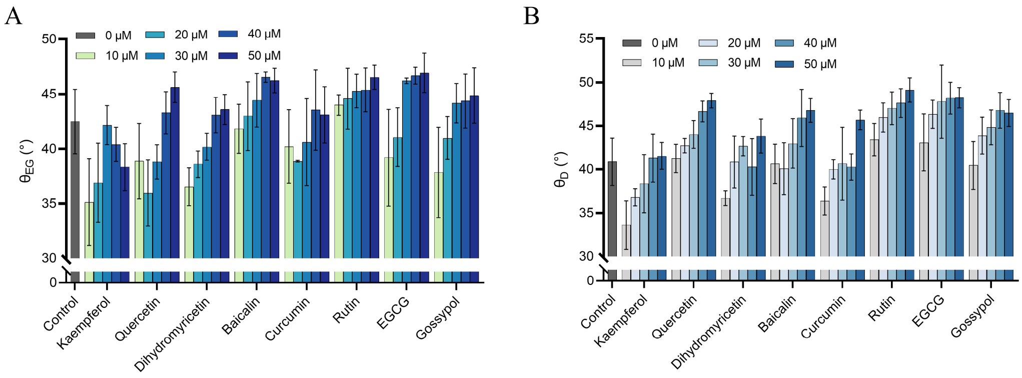

Figure S5. Static contact angles of ethylene glycol and diiodomethane measured on BAX/Bcl-2 substrate surfaces before and after polyphenols treatment.

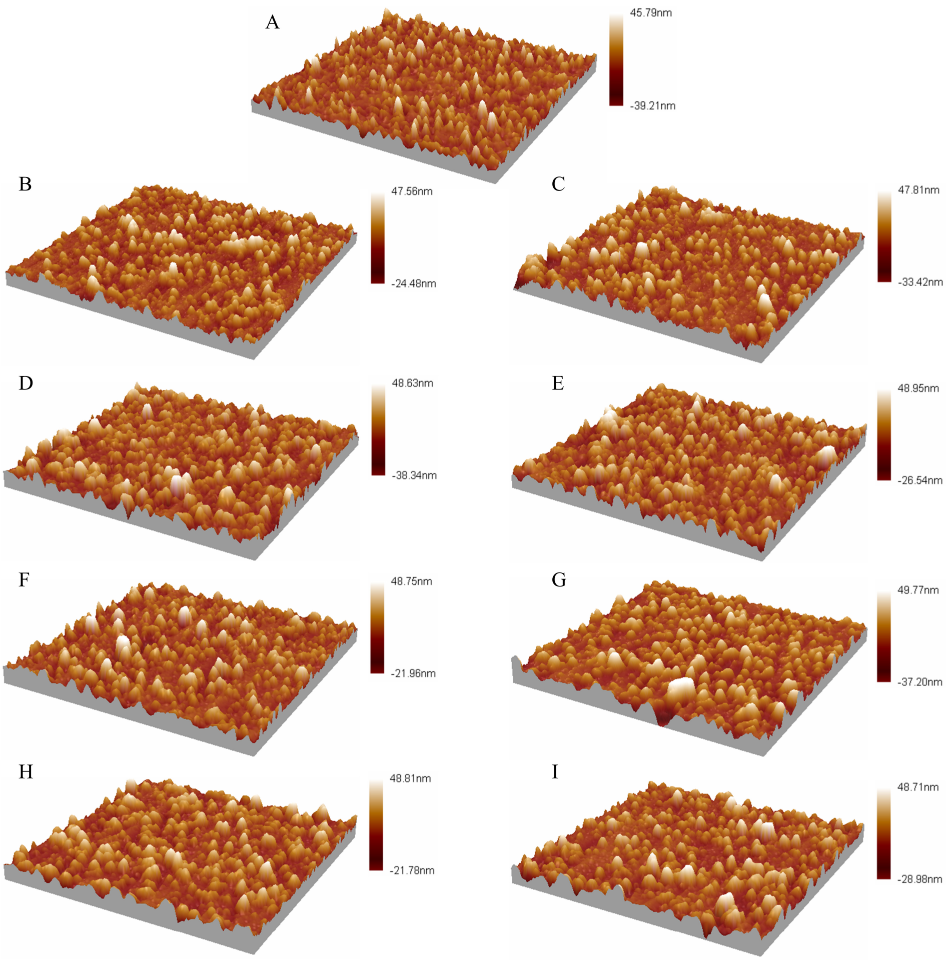

Figure S6. 3D topographies of Bcl-2 imaging in different polyphenol solutions. Bcl-2 was incubated with baicalin, epigallocatechin gallate, curcumin, kaempferol, quercetin, rutin, dihydromyricetin, and gossypol, respectively. The concentration of polyphenols was 30 μM. The scanning range was 3 × 3 μm.
